# Advancing Interprofessional Primary Health Care Services in Rural Settings for People with Chronic Low Back Disorders: Protocol of a Community-Based Randomized Controlled Trial

**DOI:** 10.2196/resprot.5914

**Published:** 2016-11-09

**Authors:** Brenna Bath, Stacey Lovo Grona, Stephan Milosavljevic, Nazmi Sari, Biaka Imeah, Megan E O’Connell

**Affiliations:** ^1^ School of Physical Therapy University of Saskatchewan Saskatoon, SK Canada; ^2^ Canadian Centre for Health and Safety in Agriculture College of Medicine University of Saskatchewan Saskatoon, SK Canada; ^3^ Department of Economics University of Saskatchewan Saskatoon, SK Canada; ^4^ Department of Psychology University of Saskatchewan Saskatoon, SK Canada

**Keywords:** low back pain, telehealth, rural health, physical therapy

## Abstract

**Background:**

Chronic low back disorders (CLBDs) are a substantial burden on individuals and societies, and impact up to 20% of Canadians. Rural and remote residents are approximately 30% more likely to have CLBDs. Reduced access to appropriate team-based health services, including physical therapy, is a key factor that may magnify the impact of CLBD on pain, physical function, overall quality of life, health-related system costs, and individual costs.

**Objective:**

The purpose of this project is to evaluate the validity, comparative effectiveness, costs, barriers, and facilitators of an interprofessional management approach for people with CLBDs, delivered via telehealth.

**Methods:**

This project will examine 3 different health care delivery options: (1) in-person nurse practitioner (NP); (2) in-person physical therapist (PT); and (3) a team approach utilizing an NP (in-person) and a PT joining via telehealth. Validity of the telehealth team care model will be explored by comparing the diagnostic categorization and management recommendations arising from participants with CLBD who undergo a team telehealth, in-person NP, and in-person PT assessment. Comparative effectiveness and costs will be examined using a community-based randomized controlled trial in a rural Saskatchewan community with limited PT services. The 3 arms of the trial are: (1) usual care delivered by a local rural NP; (2) a local NP and an urban-based PT joining via telehealth; and (3) face-to-face services by a PT traveling to the community. Patient-reported outcomes of pain, physical function, quality of life, satisfaction, and CLBD care-related costs will be evaluated up to 6 months after the intervention. Patient and provider experiences with the team telehealth approach will be explored through qualitative interviews.

**Results:**

The study was funded in July 2013 and the University of Saskatchewan Biomedical Research Ethics Board approved the study in November 2013. Participant recruitment began in September 2014 and data collection was completed in December 2015. Analysis is in progress and results are anticipated in 2017.

**Conclusions:**

CLBD is a widespread public health problem, particularly in rural and remote areas, which requires new innovative approaches to deliver appropriate health care. The results of this project will inform the development of evidence-informed approaches and community-based implementation strategies to improve access to PT services in primary health care settings in other rural and remote underserved areas. Findings might also provide a framework for cost-effective and patient-centered models of service delivery for the management of other chronic conditions.

**ClinicalTrial:**

ClinicalTrials.gov NCT02225535; https://clinicaltrials.gov/ct2/show/NCT02225535 (Archived by WebCite at http://www.webcitation.org/6lqLTCNF7)

## Introduction

Chronic low back disorders (CLBDs) are the leading cause of morbidity worldwide compared to 289 other diseases and conditions, when considering years lived with disability [1]. CLBDs are not only costly to individuals, but also strain health care resources due to increased primary physician care visits [2,3], specialist consultations, and diagnostic procedures [4,5]. Limited access to appropriate care at a primary care level is thought to be a contributing factor to this “medical disaster” [6]. Physical therapists (PTs), whose specialized knowledge of musculoskeletal conditions may exceed that of many physicians (with the exception of orthopedic surgeons [7]), have much to offer for improving the appropriateness and effectiveness of CLBD care.

Approximately 20% of Canadians report having CLBD, and people living in rural and remote regions are approximately 30% more likely to report having CLBD than their urban counterparts [8]. Lack of access to appropriate health care is thought to be a contributing factor to a higher proportion of rural individuals with chronic health conditions like CLBD, compared with urban dwellers [9,10]. However, recruitment and retention of a variety of health care providers to rural and remote regions represents a challenge to providing access to appropriate services that may help to reduce these health disparities [11]. Lack of access to appropriate CLBD care in primary health care is exacerbated in many rural and remote communities in Canada due to a general paucity of PTs [12]. For example, approximately 33% of residents in the Canadian province of Saskatchewan live in rural areas [13]; however, only 10% of the PT workforce is employed in rural communities [14].

Back pain is a common reason for seeking care at the primary care level. Jordan et al found that a quarter of all consultations in a United Kingdom physician-based primary care setting were for musculoskeletal problems, with the low back (14%) being the most common reason [3]. Back pain is also the fifth most common reason for all physician visits in the United States [15], and Canadians with chronic back disorders are 65% more likely to seek care from a family physician than those without chronic back disorders [16]. Although family physicians are often the first clinical contact for patents with low back disorders, they may not be the most appropriate health care providers to assess and treat these conditions, due to low levels of training and low perceived competence in the area [17,18]. Less than 3% of all curriculum hours in Canadian medical schools are devoted to training related to the entire musculoskeletal system, including low back disorders [19], and 82% of recent medical school graduates failed to demonstrate basic competency in assessment and management of musculoskeletal disorders [20]. Despite this low level of training, examination and treatment of low back disorders is rated by family physicians to be of significant importance, while remaining one of the lowest areas of their perceived professional competency [21]. Conversely, experienced PTs are highly competent in the assessment, diagnosis, and management of musculoskeletal disorders, including CLBDs [22,23]. Furthermore, the inclusion of PT services in primary care models for the management of low back disorders is potentially more cost-effective than family physician services alone. A systematic review found that the addition of activities (ie, education, exercise, behavioral counseling, and spinal manipulation) to usual general practitioner/family physician care for low back disorders was more cost-effective than usual general practitioner care alone [24].

In response to a shortage of family physicians in many rural and remote communities, nurse practitioners (NPs) have taken on an important role in the delivery of primary health care services [25]. NPs are advanced-practice registered nurses (usually with master’s degrees) who are able to autonomously diagnose disorders, prescribe medications, order and interpret diagnostic tests, and perform specific clinical procedures, and have been shown to provide comparable care to family physicians [26,27]. The combination of PTs with NPs for patient-centered collaborative management is a novel approach that has the potential to improve access to appropriate health care for people with CLBDs, result in improved patient outcomes, and improve overall health system efficiency for CLBD management. However, to our knowledge, similar models have not yet been developed or evaluated in the context of rural health service delivery for people with CLBDs. Additionally, the most effective and efficient means of including physical therapy services in rural health care models has yet to be explored.

The use of secure videoconferencing/telehealth is a promising means to help improve access to physical therapy services in rural primary health care settings [28]. Although videoconferencing is effective for conducting a patient interview [29], performing an effective physical examination via this medium is perceived by many clinicians to be a primary barrier in the adoption of remotely delivered services [30]. The *crux of the issue* is that elements of a conventional face-to-face physical examination require the PT to be *hands-on* with the patient [30]. Previous research has validated some components of a PT assessment via telehealth, in comparison to in-person usual care (ie, history and subjective examination) [30]; however, remote diagnosis requires a clinician to integrate the information from a detailed history and physical examination. Based on this issue, a novel approach is required to overcome the traditional barriers associated with the need for a *hands-on* assessment. An interprofessional assessment performed by an urban-based PT (collaborating via secure videoconferencing/telehealth) with a local rural NP who can perform relevant portions of the *hands-on* assessment with a rural patient with CLBD, may be a viable solution to overcome the barriers of performing an effective remote examination and allow for the development of appropriate management/educational strategies. Prior to widespread adoption of such a model, several issues need to be explored. First, the validity, comparative effectiveness, and relative costs of an electronic health model of interprofessional care, compared to face-to-face care by a PT or usual care by an NP, is unknown and has yet to be developed or examined. Second, the impact of videoconferencing on clinical workflow practices and interprofessional collaboration is an area that requires further study [31]. Finally, understanding readiness within rural and remote communities is an important step for the successful implementation and sustained use of videoconferencing-type services in existing systems of health care [32].

The objectives of this research study were to: (1) explore the *validity* (ie, diagnostic and management concordance) of an interprofessional assessment session with a PT and NP performed via secure videoconferencing, compared to a PT or NP in-person assessment alone; (2) examine the *impacts* and *cost-effectiveness* of an interprofessional assessment/education session with a PT and NP delivered via secure videoconferencing for people with CLBDs, compared to in-person PT only assessment/education session and usual care by an NP; and (3) explore the *perceived barriers and facilitators* of the use of secure videoconferencing for assessment and management of people with CLBDs living in rural underserved communities, from the perspectives of patients and health care providers.

## Methods

### Operational Definition of Chronic Low Back Disorder

Low back disorders include a large group of clinical and etiological entities and there is no *gold standard* clinical classification or diagnostic criteria for many of these conditions [33]. Furthermore, the International Classification of Diseases-10 system does not have an adequate and distinct diagnostic code(s) for chronic pain or CLBD [34]. Thus, for this study, CLBDs include self-reported pain and disability that has lasted for a minimum of 3 months that is related to low back injury (ie, sprain/strain), and/or low back pain, and/or associated hip or leg symptoms due to pain referral. CLBDs may develop from trauma or, more often, from repetitive or cumulative loading mechanisms that lead to adverse structural changes in spinal soft tissue and articular structures [35], which often have chronic, episodic, or recurrent manifestations [36].

### Secure Videoconferencing Platform and Procedures

The secure videoconferencing platform used was VidyoDesktop software (Vidyo Inc., Hackensack, NJ, USA) installed on laptop computers. A detachable external web camera with remote pan, tilt, and zoom functions was attached to the laptop in the *rural* location (ie, with the NP and participant). If required, the NP could use the remote to direct the camera to provide different views of the participant during the physical examination, as directed by the PT.

### Study Setting, Population, and Recruitment Strategies

The validity part of the study (Objective 1) took place in the city of Saskatoon, Saskatchewan (SK). The rural pilot trial (Objectives 2 and 3) took place in the Kelsey Trail Health Region in the communities of Arborfield and Carrot River (264 to 288 kilometers from Saskatoon, SK, respectively). These communities were identified through a combination of related research, an environmental scan, and consultation with local health care providers and managers as having reduced access to local PT services. The Kelsey Trail Health Region has an estimated population of 40,000 people and 32.2 PTs per 100,000 residents (a total of 13 PTs in 2010) compared to 83.2 PTs per 100,000 residents (a total of 262 PTs) in the Saskatoon Health Region [37]. The 2 communities are within 24 kilometers of each other and are served by 3 NPs (2 in Carrot River and 1 in Arborfield). The estimated caseload proportion of patients seeking care with CLBDs in these practices is 20% (personal communication, Kowal L, December 2012) which is consistent with published literature [3,15]. People aged 18 to 80 years with pain and discomfort localized below the costal margin and above the gluteal folds, with or without leg pain, that (1) limits usual activities or daily routine and (2) has been present for more than months [38] were invited to participate for each part of the study. Exclusion criteria included: people currently receiving third party payer funding (ie, Worker’s Compensation Board, or other) for their back-related complaints; people with primarily neck (cervical spine) or mid-back (thoracic spine) complaints; and people with language, reading, or comprehension barriers that would limit adequate completion of the study paperwork. Recruitment strategies included advertisements in local newspapers, provision of study details when presenting for care at any of the participating rural providers, and posters posted at health care facilities and other community centers.

### Study Design and Data Collection

Concurrent to the intervention part of the study described below, 30 people with CLBDs were recruited from the Saskatoon, SK area. Each participant underwent an interprofessional assessment with an NP (in-person) and a PT (via videoconferencing), an in-person assessment with a second PT, and an in-person assessment with a second NP. Each PT and NP completed an online clinical classification tool, adapted from one previously developed by the author [39], to allow for an interrater comparison of diagnostic and management recommendation classification (ie, PT in-person vs NP in-person vs team of NP in-person with PT joining via secure videoconferencing).

**Figure 1:**
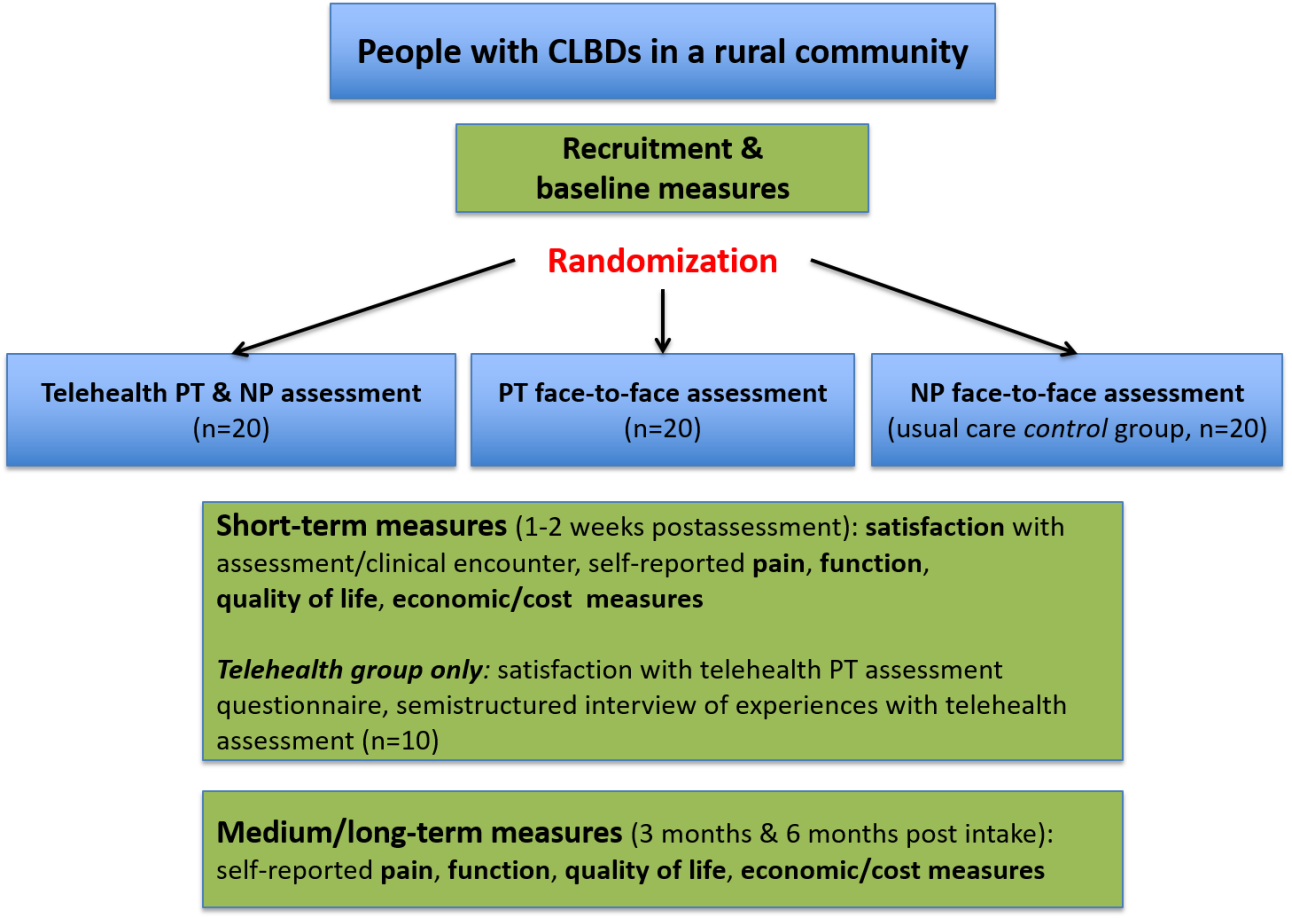
Design of rural community-based trial part of the study. CLBD: chronic low back disorder; NP: nurse practitioner; PT: physical therapist.

The study design for Objectives 2 and 3 included two intervention groups and a control group, with 20 participants in each group: (1) interprofessional telehealth intervention; (2) in-person PT (travelling from Saskatoon, SK to provide services); and (3) *usual care* provided by an NP (see Figure 1). Due to the interprofessional nature of the intervention, the NP involved in the telehealth-based intervention group may have altered their *usual care* practice; therefore, the control group participants were drawn from the practices of 2 NPs that were not involved in the telehealth intervention, who provided services out of Carrot River. Participants were randomly assigned to 1 of the 3 groups using simple block randomization to ensure equal group sizes [40]. Participants allocated to either of the in-person PT group or the team telehealth intervention groups were eligible to receive up to 4 in-person PT treatment sessions delivered by an urban-based PT who travelled to the community (if recommended by the assessing health care providers).

A combination of paper-based and online questionnaires measured outcomes at 4 time points, as shown in Figure 1: (1) *baseline*, prior to the health care encounter; (2) *short term*, within 2 weeks of the initial health care encounter; (3) *medium term*, 3 months postintake; and (4) *longer term*, 6 months postintake. Baseline questionnaires covered a range of sociodemographic (eg, age, gender, education, employment and other income, work status), clinical (eg, pain location, duration) and psychological (eg, fear avoidance beliefs, depression, somatization) factors. Phone or email reminders for completion of follow-up questionnaires were performed with phone or email prompts based on the tailored design method proposed by Dillman et al [41].

The multidimensional outcome measures have demonstrated reliability, validity, and responsiveness in similar clinical populations, and will cover the domains of back-specific function, general well-being/generic health status, pain, work disability, and satisfaction with care, as recommended by international groups of back pain researchers [42,43]. The primary outcome of interest will be self-perceived function, which was assessed using the modified Oswestry Disability Index, a back-specific self-report questionnaire [44,45]. The Numeric Pain Rating Scale [46] was used to measure the intensity of current pain, pain at its best, and pain at its worst levels over the last 24 hours. Quality of life/general health status was measured with the EuroQol health survey instrument (EQ-5D-5L) [47]. Patient satisfaction was measured using a modified version of the Visit-Specific Satisfaction Instrument, as described and validated by Kennedy et al [48], as well as a space for comments regarding satisfaction with the clinical encounter, as previously published [49]. Costs were captured using self-report diaries that recorded intervention/treatment costs, work status, absenteeism and disability days related to back pain, health service use within and outside of the study (ie, both government funded and nonfunded services), and other CLBD-related costs such as medication use (ie, prescription and nonprescription drugs) and travel time and costs from the beginning of the intervention period until the end of the study period (ie, 6 months postintake).

Exploration of perceived barriers and facilitators regarding the implementation and use of secure videoconferencing was undertaken using surveys of participants of the intervention group, and the NPs and PTs involved in the videoconferencing intervention group. An adaptation of a tool developed by Russell et al [30] to measure satisfaction with PT-delivered telehealth assessments was administered to participants in this group at the short-term follow-up period. Six patient participants were invited to participate in a 30-minute semistructured interview within 2 weeks of their initial assessment date.

### Analyses

Analyses are currently in process and not yet complete.

#### Clinical Validity of Team Telehealth Assessment

Descriptive statistics will be calculated to examine select demographic and clinical characteristics of the study sample. Differences in these variables between participants comprising the subset for the validity part of the study (n=30), and the participants in the intervention part of the study (n=60), will be evaluated with independent samples *t*-tests and chi-square tests. The level of agreement for diagnostic and management categories between each provider group will be calculated with the kappa coefficient. Weighted kappas will be calculated for categories in which more than 2 options are possible [50]. Overall observed agreement (ie, proportion of cases for which the providers agreed) will also be calculated.

#### Impacts and Cost-Effectiveness of Rural Trial

Descriptive analyses of all baseline measures will include frequencies and valid percent for categorical variables, and mean, standard error, median, and interquartile ranges for continuous variables. Comparisons between the baseline and outcomes at short-, medium-, and long-term time points will be completed with parametric tests (eg, paired *t*-tests) or nonparametric test equivalents (eg, Mann-Whitney *U*) where appropriate. The primary outcome will be the Oswestry Disability Index. Multivariable regression analysis (as described by Salisbury et al [51]), conducted on an intention-to treat basis, will be used to investigate between-group differences in mean Oswestry Disability Index scores at the 6-month time point, with adjustment for baseline scores. The economic evaluation will be conducted using the cost utility analysis. This analysis will involve the use of the EQ-5D-5L health survey instrument [47], for which utility weights are available for a sample of the Canadian general population [52]. Multiple linear and logistic regression analyses will be used to determine the predictive models that best explain differences and changes in both outcomes and costs related to both productivity loss and health care costs.

#### Perceived Barriers and Facilitators for Videoconferencing

An inductive thematic analysis will be applied to qualitatively analyze the results of the semistructured interviews with the PTs and NPs involved in the team telehealth assessment, and a sample of patient participants from the team telehealth intervention arm. A process of open and axial coding will be applied. During open coding, a constant comparative approach will be used to group the codes into categories and identify themes. Axial coding will then be completed to look at the interrelationship of categories. A coding scheme will be developed jointly, and verified independently, by 2 researchers via identifying, classifying, and labeling the primary patterns in the data.

### Sample Size

A sample size of 30 for Objective 1 (validity) is adequate, based on an estimated minimum .60 kappa level between 2 PT raters and 80% power [50]. Determining the appropriate sample sizes to reach adequate power for Objectives 2 and 3 (rural community trial) is challenging, as there has been little comparable work in similar clinical populations from which to draw estimates of variance and effect sizes. Furthermore, the sample sizes suggested in the literature [46] far exceed what would was feasible given the budget, time lines, and caseload proportion of CLBDs in the rural community. A primary purpose of the intervention part of this study will be to estimate outcome variances and effect sizes, which could then be used to plan a larger, sufficiently powered, multi-site intervention.

### Ethical Considerations

The largest potential burden for participants was perhaps the amount of time required to complete the baseline and follow-up questionnaires (30-60 minutes). Some participants may have felt sensitive about recording psychosocial risk factors or health history; however, assurances of study data confidentiality and anonymity should have helped to address this. All study protocols complied with Health Information Protection Act standards. The VidyoDesktop platform is a private and secure means of sharing sensitive health and personal information between patients and health care providers. Consent forms were reviewed and signed by participants prior to starting the study. Study participants’ data will be confidential and identified only by study identification number; participants will not be identified in any reporting materials and only aggregate data will be presented during results dissemination. All data will be stored on a password-protected server at the University of Saskatchewan.

### Knowledge Translation

The proposed project will implement the Canadian Institutes of Health Research’s integrated knowledge translation approach, engage researchers and knowledge users throughout the research process, and maximize prospects for the use of findings in practice [53]. An array of clinical, community-based, and manager/decision maker knowledge users have been (and will continue to be) recruited to participate in this study, thereby shaping its design and driving health care practice implications. These partners will be consulted and engaged at key stages throughout the research process (ie, 1-2 times per year through a combination of in-person and teleconference/videoconference meetings over the course of the 3-year project).

## Results

This study was funded in July 2013 and the University of Saskatchewan Biomedical Research Ethics Board approved the study in November 2013. Participant recruitment began in September 2014 and data collection was completed in December 2015. Analyses are in progress, and results are anticipated in 2017. Results of the trial component of this study will comply with the CONSORT-EHEALTH checklist [54].

## Discussion

CLBDs are a widespread public health problem, particularly in rural and remote areas. New innovative models of care delivery are needed to address reduced access to PT services in many rural and remote communities worldwide. The aim of this project is to compare *usual care* delivered by a rural health care provider (ie, NP) with 2 means of integrating a PT into a rural primary health care setting: (1) PT joining via secure videoconferencing/telehealth for a team-based approach with the in-person NP; and (2) the PT travelling from an urban center to provide face-to-face services in a rural community. This study has the potential to inform rural and primary health care reform in Saskatchewan and beyond, to improve access to needed health services in underserved rural communities, and lead to the development of important partnerships that will lay the foundation of a Saskatchewan-based program of research. The lessons learned from this project regarding barriers and facilitators will help to inform effective strategies for implementation and evaluation of similar care models in different rural and remote communities and health care contexts. This research may also help to optimize management of a range of common chronic conditions in rural and remote settings.

Despite the novel contribution of this study to the literature, and its potential to inform health services reform and access, there are notable limitations. First, given the complex and heterogeneous nature of CLBDs and variability in the biopsychosocial experiences of those with CLBDs, the characteristics of participants in this study may not be reflective of the broader population with similar conditions. Second, the assessment and management of CLBD is similarly complex and heterogeneous. Although the primary aim of this study is the comparison of a team telehealth/videoconferencing approach to in-person PT or NP care, we anticipate wide intraprofessional (as well as interprofessional) variability in approaches, which may limit the replicability of the study intervention in other contexts and with different health care providers. Countering these limitations are the use of multidimensional outcome measures using both qualitative and quantitative approaches, which will allow for a more nuanced evaluation of the different care models. Unfortunately, this study does not include an in-person *team* approach (PT and NP) as one of the comparison groups. This omission is mainly due to scope limitations imposed by funding availability. Further research should examine the validity, feasibility, and impacts of this additional health delivery model for people with CLBDs. The current research is predominantly a pilot and feasibility study, but we anticipate that the findings and lessons learned from this study will nevertheless be valuable to inform future research and health services planning.

This study is the first step of a planned multi-stage research program in which future studies will investigate how similar interventions may work in different rural and remote communities, and with other types of chronic conditions and populations. The results of this project will lead to the development of evidence-informed approaches and community-based implementation strategies to improve access to PT services in primary health care settings in other rural and remote underserved areas, and potentially provide a framework for cost-effective and patient-centered models of service delivery for management of other chronic conditions. Furthermore, the partnership approach to health services research is crucial to lay the foundation for the development and evaluation of more extensive multi-site, community-informed, team-based interventions for people with CLBDs and other chronic health conditions living in rural and remote communities.

## Abbreviations

CLBD: chronic low back disorder
EQ-5D-5L: EuroQol health survey instrument
NP: nurse practitioner
PT: physical therapist
